# Intestinal microbiota as a tetrahydrobiopterin exogenous source in *hph-1* mice

**DOI:** 10.1038/srep39854

**Published:** 2017-01-12

**Authors:** Jaques Belik, Yulia Shifrin, Erland Arning, Teodoro Bottiglieri, Jingyi Pan, Michelle C. Daigneault, Emma Allen-Vercoe

**Affiliations:** 1 Physiology & Experimental Medicine Program, The Hospital for Sick Children Research Institute, Toronto, Ontario M5G 1X8, Canada; 2 Department of Paediatrics and Physiology, University of Toronto, Toronto, Ontario, M5G 1X8 Canada; 3 Baylor Research Institute, Institute of Metabolic Disease, Dallas, TX, 75226, USA; 4 Molecular and Cellular Biology, University of Guelph, Guelph, Ontario, N1G 2W1 Canada

## Abstract

Tetrahydrobiopterin (BH4) is a cofactor of a number of regulatory enzymes. Although there are no known BH4 exogenous sources, the tissue content of this biopterin increases with age in GTP cyclohydrolase 1-deficient hyperphenylalaninemia-1 (*hph-1*) mice. Since certain bacteria are known to generate BH4, we hypothesize that generation of this biopterin by the intestinal microbiota contributes to its tissue increase in *hph-1* adult mice. The goal of this study was to comparatively evaluate *hph-1* mice and wild-type C57Bl/6 controls for the presence of intestinal BH4-producing bacteria. Newborn and adult mice fecal material was screened for 6-pyruvoyltetrahydropterin synthase (PTPS-2) an enzyme only present in BH4-generating bacteria. Adult, but not newborn, wild-type control and *hph-1* mouse fecal material contained PTPS-2 mRNA indicative of the presence of BH4-generating bacteria. Utilizing chemostat-cultured human fecal bacteria, we identified the PTPS-2-producing bacteria as belonging to the Actinobacteria phylum. We further confirmed that at least two PTPS-2-producing species, *Aldercreutzia equolifaciens* and *Microbacterium schleiferi*, generate BH4 and are present in *hph-1* fecal material. In conclusion, intestinal Actinobacteria generate BH4. This finding has important translational significance, since manipulation of the intestinal flora in individuals with congenital biopterin deficiency may allow for an increase in total body BH4 content.

Tetrahydrobiopterin (BH4) is an important regulatory enzyme cofactor for the catecholamine, indoleamine and nitric oxide synthase biochemical pathways. A reduced or absent BH4 endogenous pool results in severe clinical manifestations such as phenylketonuria, movement disorders, systemic and pulmonary hypertension^1^,^2^,^3^.

The hyperphenylalaninemia-1 (*hph-1)* mouse strain was created via *N*-ethyl-*N*-nitrosourea (ENU) mutation and has a partial deficit in GTP cyclohydrolase I (EC 3.5.4.16; GTPCH1), the first and rate-limiting enzyme responsible for *de novo* BH4 generation^4^. When compared with wild type control animals, adult *hph-1* mice have a higher systemic blood pressure^1^ and manifest pulmonary hypertension as early as the neonatal period^5^.

Interestingly, the *hph-1* mice tissue BH4 content is age-dependent. GTPCH1 activity of adult *hph-1* mice is two-fold higher when compared with the newborn counterpart^6^,^7^. We previously reported, however, that newborn *hph-1* mouse lungs have 30-fold lower tissue BH4 content, when compared with same age wild-type controls^5^. Thus, age-related GTPCH1 activity changes cannot fully account for the much higher adult *hph-1* mice BH4 tissue content.

Of clinical relevance is the fact that the age-dependent tissue BH4 content pattern may account for certain phenotypic changes that are only present early in life in these mice. For instance, idiopathic hypertrophic pyloric stenosis is present in 1–2 week old *hph-1* mice, but completely regresses in adult life^8^. BH4 deficiency has also been linked to the idiopathic human condition, hypertrophic pyloric stenosis, through a mechanism based on nitric oxide synthase-dependent pyloric sphincter regulation^8^. Idiopathic hypertrophic pyloric stenosis in humans solely manifests in infants and if left untreated, the condition spontaneously resolves^9^.

Aside from the *de novo* pathway, BH4 is generated via the so-called salvage pathway^2^ through recycling via dihydrofolate reductase of its oxidized product, dihydrobiopterin (BH2). This pathway, however, cannot account for the BH4 tissue levels in adult *hph-1* animals since these animals have reduced BH2 as a substrate for recycling. It is, therefore, possible that *hph-1* mice are able to obtain BH4 in adult life via exogenous sources, yet this biopterin is not present in food.

The intestinal microbiota has been recently gaining a lot of attention as an enteral source of metabolites that contribute to the endogenous pool^10^,^11^,^12^. Cyanobacteria such as *Synechococcus elongatus (S. elongatus),* depend on pterins for pigment color generation and contain 6-pyruvoyltetrahydropterin synthase (EC 4.2.3.12; PTPS) an enzyme only present in BH4-generating bacteria^13^ and regulated by the PTPS-2 gene. We hypothesized that the intestinal microbiota also contains bacteria capable of generating BH4, and that PTPS-2 expression could be used as a biomarker for this activity. We therefore screened fecal material from wild type and *hph-1* mice for the presence of BH4-generating bacteria. We then went on to screen representative human microbial communities for PTPS-2 expression. We present data demonstrating that adult mice and human intestinal microbiota contain bacteria capable of BH4 generation.

## Results

### Tissue BH4 content

**Figure 1:**
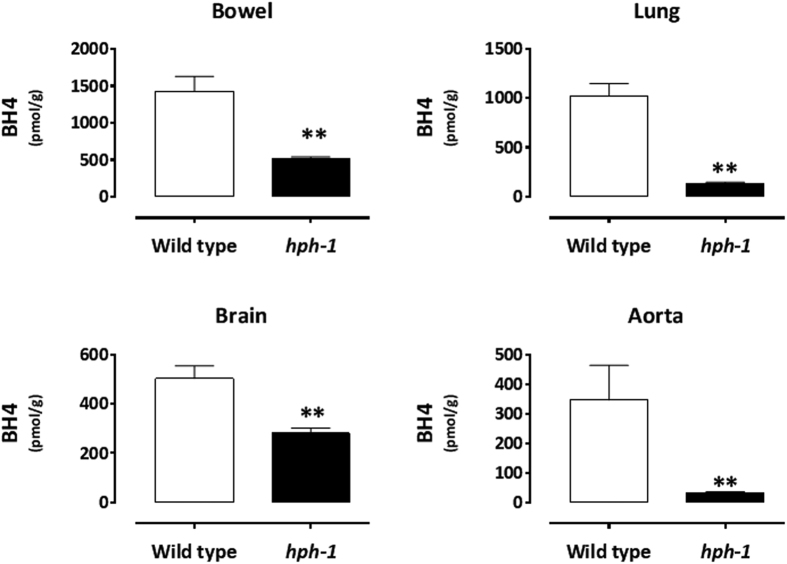
Adult wild type and *hph-1* bowel, lung, brain and aorta BH4 tissue content. N = 4 for all strain/organ groups. **P < 0.01 when compared with wild type group by Student unpaired t-test.

We first conducted a comparative analysis of adult *hph-1* and wild type control mice BH4 tissue content. As shown in Fig. 1, when compared with wild type animals, the *hph-1* mouse large intestine (bowel), lung, brain and aorta BH4 tissue content was significantly reduced (P < 0.01), although this biopterin was present in all measured tissues.

### Fecal material PTPS-2 mRNA expression

**Figure 2:**
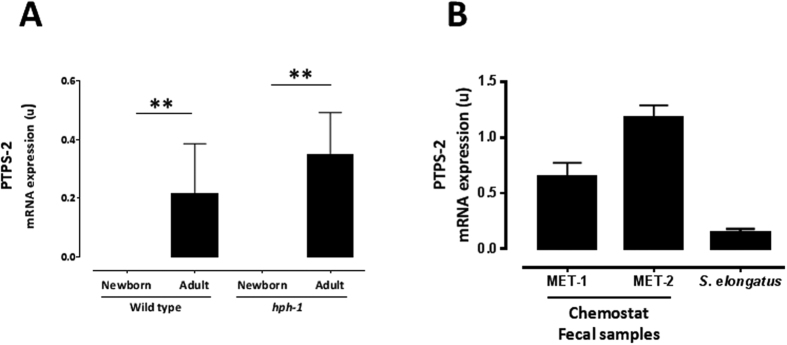
6-pyruvoyl tetrahydropterin synthase 2 (PTPS-2) mRNA expression from wild type and *hph-1* newborn and adult animals’ fecal colonic material (Panel A; N = 6 per strain/age) and MET-1 and MET-2 chemostat fecal samples (Panel B; N = 3 per group). *S. elongatus* was used as a positive control. **P < 0.01 by Student unpaired t-test.

We proceeded to screen colonic material from newborn and adult mice for the presence of PTPS-2. PTPS-2 mRNA expression was detectable in wild type and *hph-1* adult mice, but absent in the newborn animals (Fig. 2A). No significant difference in PTPS-2 mRNA expression was observed between the mouse strains.

### Identification of the PTPS-2 containing intestinal bacteria

We used diverse, defined pools of bacterial isolates from human feces that were cultured as communities in chemostat bioreactors^14^,^15^ to evaluate the presence of PTPS-2 expressing bacteria. As shown in Fig. 2B, PTPS-2 mRNA expression was detected in the two distinct chemostat fecal microbiota cultures, MET-1 and MET-2.

To identify the PTPS-2 expressing bacteria species in the chemostat-cultured fecal material, we first divided the communities up by phyla and evaluated each of these groups individually. The strains expressing PTPS-2 are illustrated in the Table 1.

**Figure 3:**
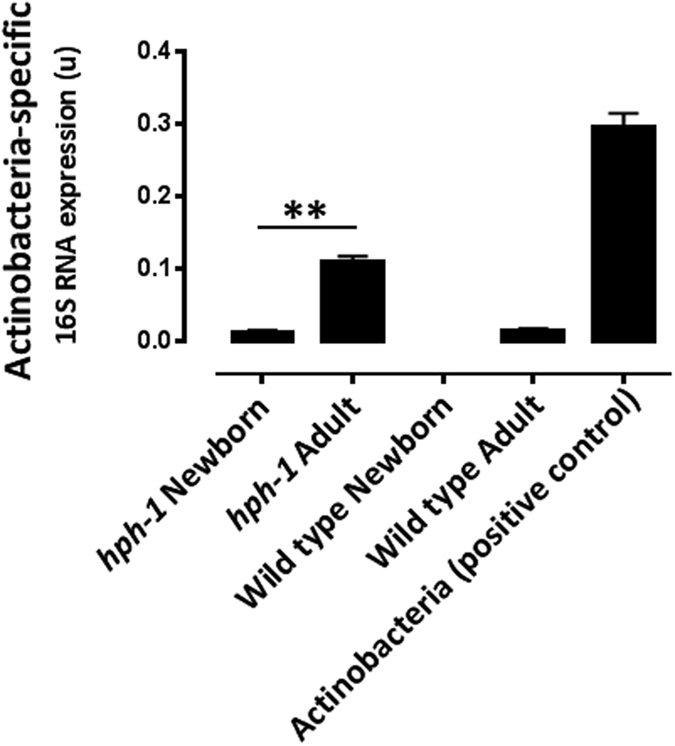
*Actinobacteria*-specific 16S rRNA screening of wild type and adult mice fecal material *Actinobacteria* was used as a positive control. N = 3 per group for the 3 panels. **P < 0.01 by Student unpaired t-test.

We next proceeded to screen colonic material from the newborn and adult mice for the presence of Actinobacteria-specific 16S rRNA, and confirmed that this bacterial phylum can be detected in adult mice (Fig. 3).

### Actinobacteria generate BH4

**Figure 4:**
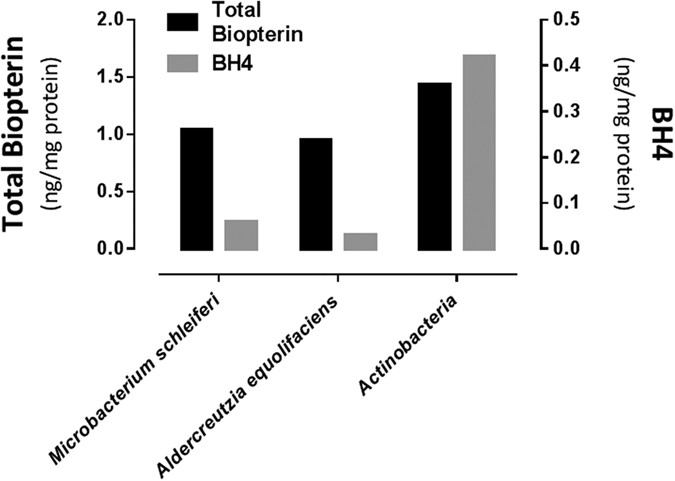
Chemostat-derived Actinobacteria and cultured *Aldercreutzia equolifaciens* and *Microbacterium schleiferi* strains total biopterin and BH4 bacteria pellet content.

We first screened adult *hph-1* mouse colonic fecal material for the presence of BH4 and determined the content of this biopterin to be 1.2 ± 0.1 ng/g of fecal material (N = 3). Using human fecal material, we next evaluated whether the identified Actinobacteria spp. are capable of generating biopterin. As shown in Fig. 4, defined, chemostat-derived total Actinobacteria (containing *Bifidobacterium longum, Bifidobacterium adolescentis, Collinsella aerofaciens, Aldercreutzia equolifaciens, Microbacterium schleiferi, and Micrococcus luteus)* generated total biopterin, and more specifically BH4, as respectively measured by HPLC and LC-MS/MS techniques. We then went on to measure BH4 in microbial extracts from the axenically grown Actinobacteria spp. from these chemostat cultures, and found that the source of BH4 was *Aldercreutzia equolifaciens* and *Microbacterium schleiferi* (Fig. 4).

### The potential for generation of BH4 by intestinal Actinobacteria is prevalent

Using a library of human feces-derived Actinobacteria spp., we went on to screen for the presence of PTPS-2 in these strains in order to assess how widespread BH4 generation ability is among this phylum. We axenically cultured 31 additional Actinobacteria isolates and assessed them for the presence of PTSPS-2 by real-time-qPCR. We found several isolates which expressed PTPS-2 (Table 1), including representatives of several species of *Bifidobacterium* that have been previously noted to be of importance to neonatal health^16^,^17^.

## Discussion

In this study, we documented the presence of BH4-generating bacteria in the mouse gut microbiota. The microbes responsible for BH4 production in the intestinal microbiota were found to belong to the Actinobacteria phylum, and we went on to show that strains of several Actinobacteria spp. derived from the human gut are able to express the PTPS-2 gene, accounting for BH4 generation in defined, chemostat-cultured, human fecal material.

*hph-1* mice show a 90% decrease in GTPCH1 expression when compared with wild-type controls^18^,^19^,^20^,^21^. This deficiency results in low to absent tissue BH4 levels at birth. The *hph-1* mouse tissue BH4 increases with age^1^,^22^ and the adult *hph-1* mouse ileal tissue BH4 content is similar to that of wild-type control animals^23^.

The partial and age-dependent GTPCH1 expression/activity in the *hph-1* mice contributes to the higher tissue BH4 content in adult *hph-1* mice, when compared with early in life. Yet other factors likely play a role in the age-related organ BH4 content changes. In the present study, we hypothesize, and provide supporting evidence, that the rodent and human intestinal microbiota are capable of generating this biopterin.

Over the last two decades, both green sulfur bacteria and cyanobacteria have been postulated as BH4 producers based on the discovery of PTPS-2 orthologs in their genomes^13^,^24^,^25^. Pteridines are involved in pigmentation and thus the biopterin generating ability of these bacteria may relate to photosynthetic requirements. However, while bacterial BH4 production ability is established, phototrophic bacteria do not colonize the intestine, and thus the extent to which the intestinal microbiota is capable of BH4 generation, and its contribution to the endogenous pool of this biopterin, to the best of our knowledge has not been adequately investigated.

Bacteria are known to generate nutritional factors that can be absorbed by the gastrointestinal tract and play important functional roles in mammals. Examples of this process include orally derived nitrate^26^,^27^ and folic acid^28^,^29^,^30^. A stable oral form of BH4 is currently in use clinically (Kuvan©) attesting to the gastrointestinal route as an efficient transporter of this biopterin^31^. It is likely, therefore, that the generation of BH4 by the intestinal microbiota contributes to its presence in adult *hph-1* tissues.

It is known that the gut microbiota modulates the development of the enteric nervous system in mice^10^. We show in the present study that *Bifidobacterium* spp., a major group of Actinobacteria within the mammalian gut microbiota, and the predominant intestinal genus in breast-fed infants^32^, express PTPS-2. In addition, we have shown that human breast milk has a high BH4 content^33^ and may thus be an important contributor to the endogenous BH4 pool early in life. In fact, idiopathic hypertrophic pyloric stenosis is rarely present in breast-fed infants^34^.

These data together with the present finding that PTPS-2 expressing bacteria were not detected in newborn mice suggest that major contributors to the BH4 endogenous pool are breast milk early in life followed by the intestinal microbiota as it becomes established into adulthood. We anticipate this may be of particular importance in subjects with inborn errors of biopterin metabolism in which BH4 synthesis has been compromised.

Although our screening for the presence of human-derived Actinobacteria spp. with PTPS-2 expression ability was limited, it is important to note that this activity does not seem to be species specific. In three cases, we noted that PTPS-2 expression could be detected for one but not another strain of the same species (*Bifidobacterium infantis, Eggerthella lenta and Gordonibacter urolithinfaciens*). This suggests that either there are differences in PTPS-2 gene presence among strains of the same species, or that gene expression is limited by environmental conditions. Further work is necessary to definitively delineate the presence of PTPS-2 genes across the Actinobacteria phylum, and to assess whether these genes are active in the mammalian intestine and responsible for BH4 production that can be utilized by the host for metabolic purposes.

This study has some limitations. Our primary goal was to identify specific bacteria capable of BH4 generation. Although we began our studies by assessing BH4 production in a mouse model, we went on to screen defined chemostat-cultured human fecal material for the presence of PTPS-2 expressing bacteria since we considered this translationally relevant to human disease. Indeed, our chemostat bioreactor model is set up to mimic conditions of the human intestine and not easily adaptable for inoculation by mouse fecal pellets. Thus, we have not assessed mouse-derived Actinobacteria in this study beyond our initial screening.

In conclusion, the intestinal microbiota contains BH4 generating bacteria and these likely contribute to the age-dependent rise in tissue levels of this biopterin in *hph-1* mice, a BH4 deficient strain. The human gut microbiota additionally contains PTPS-2-expressing bacterial species within the Actinobacteria phylum. Our findings have important translational significance, since manipulation of the intestinal flora may significantly influence the total body BH4 content, and this may be therapeutically useful in cases of BH4 insufficiency.

## Methods

### Chemicals and reagents

Standards for (6R, S)-5,6,7,8-tetrahydrobiopterin (BH4) and L-7,8-dihydrobiopterin (BH2) and labeled stable isotope internal standards ^15^N-BH4 and ^15^N-BH2 were obtained from Schircks Laboratories (Switzerland). Optima LC-MS grade methanol was obtained from Fisher Scientific (Ottawa, ON, Canada) and L-biopterin from Cayman Chemicals (Burlington, ON, Canada). Stock solutions of each pterin analytical standard and stable isotope were prepared as 1 mmol/L in water containing 0.2% dithiothreitol (DTT) and aliquots were stored at −80 °C. All other chemicals and reagents were obtained from Sigma Aldrich (Oakville, ON, Canada).

### Animals

All procedures were conducted in agreement with the Canadian Animals for Research Act (1990) Canadian Council on Animal Care (CCAC) regulations, and the study protocol was approved by the Hospital for Sick Children’s Animal Care Committee. Adult *hph-1* mice were bred in-house and genotyped to confirm homozygous, dominantly-inherited GTPCH1 gene deficiency (data not shown). C57Bl/6 mice (Charles River, ON, Canada) were utilized as wild type controls, since this is the background of the *hph-1* mice utilized in this study.

All animals were fed regular rodent pellets (Ren’s Feed & Supply Ltd. Aberfoyle, ON, Canada) and housed under standard lighting and temperature conditions. Newborn (<8 days of age) and adult (>60 days old) wild-type and *hph-1* mice of both sexes were studied. The animals were killed through cervical dislocation (newborn), or pentobarbital sodium (60 mg/kg ip - adult) and tissue from several organs was quickly excised, snap-frozen in liquid nitrogen and stored for later processing. The large bowel was isolated for fecal material collection and subsequently snap frozen for further processing.

### Actinobacteria culture

For assessment of PTPS-2 gene presence among Actinobacteria, selected Actinobacteria strains (all originally isolated from human sources) were cultured at 37 °C on Fastidious Anaerobe Agar (FAA, Acumedia, Lansing, MI) supplemented with 5% (v/v) defibrinated sheep’s blood (Hemostat Laboratories, Dixon, CA). All incubations were carried out anaerobically, except for NEC1 FAA aer, 22-5-S 1 D5 FAA aer, 32-6-I 1 BHI aer and NEC1 5 TSA aer, which were cultured in air. For each strain, isolated colonies were selected, resuspended in Tris-EDTA, and subjected to DNA extraction. Briefly, cells were broken open by first incubating each sample with 0.2 mg proteinase K and 0.5 mg lysozyme (Sigma Aldrich) for 1 hr at 37 °C, bead–beating with glass beads at 3000 rpm for 4 minutes, and then heating to 90 °C for 10 minutes. Broken cells were then subjected to DNA extraction using the Promega Maxwell 16 instrument according to manufacturer’s instructions. Extracted, purified gDNA was subjected to real-time q-PCR analysis as described below.

### Tissue and bacteria pellet BH4 analysis

Determination of BH4 in tissue and bacterial pellets was performed by LC-MS/MS as previously described^35^ with modifications to sample preparation. Briefly, analysis was performed on an AB Sciex 5500QTRAP mass spectrometer (Foster City, CA, USA) coupled with a Shimadzu Nexera ultrahigh pressure liquid chromatograph system (Kyoto, Japan). Pterins were separated by a binary gradient using reversed-phase HPLC on an EZfaast 250 × 2 mm 4 μm AAA-MS column, with a 4 × 2 mm Security Guard column. A calibration curve was prepared in water containing 0.2% dithiothreitol (DTT) (Sigma Aldrich, Oakville, ON) over the range of 25–1600 nmol/L for BH4. A deproteinization solution containing internal standards for ^15^N-BH4 was prepared in 0.1 M perchloric acid (Sigma Aldrich) containing 0.2% DTT at a final concentration of 1000 nmol/L each.

Tissue sample preparation involved deproteinizing at a 1:5 or 1:10 dilution with deproteinization solution and homogenizing with pestle using an overhead stirrer. Following deproteinization the tissue sample was centrifuged at 14800 rpm at 4 °C for 10 minutes. 30 μl of supernatant was combined with 120 μl of water containing 0.2% DTT. Processed supernatants were transferred to a microtiter plate and 10 μL was injected for analysis.

Prepared cell pellets were separately resuspended in 100 μL of 10 mM Tris-HCl, pH 8.0, 1 mM EDTA. After sonication (1 minute), each homogenate was separately mixed with an equal volume of acidic iodine solution (2% KI, 1% I_2_ (w/v) in 1 N HCl). After vortexing, the mixture was left in the dark for 1 hour at room temperature and then centrifuged at 20,000 g for 15 minutes at 4 °C. The supernatants were mixed with one-tenth volume of 5% (w/v) ascorbic acid to reduce excess iodine and assayed.

### Real-time-qPCR of intestinal microbiota

Genomic bacterial DNA was isolated from the stored newborn and adult wild type and *hph-1* mice fecal colonic matter, and defined chemostat-cultured human fecal material using Stool DNA isolation kit (Norgen Biotek, Thorold, ON, Canada) according to manufacturer’s instructions. DNA from bacterial cell pellets was isolated using Quick-gDNA MiniPrep kit (Zymo research, Irvine, CA, USA). The DNA obtained was stored at −80 °C. PTPS-2 genes were amplified from the bacterial pellet by quantitative PCR (ABI Prism 7900; Applied Biosystems by Life Technologies, Carlsbad, CA, USA) using SYBR Select master mix (Life Technologies, Carlsbad, CA, USA). *Synechococcus elongatus (S. elongatus)*, a known BH4-producing bacterium, was used as a positive control. Bacterial PTPS-2 expression was evaluated using the following primers: PTPS-2 S: 5′- ATGAGAGACAGCCAATCACG-3′; PTPS-2 AS: 5′- TTAGAGCAAAACGGGTACTG-3′. Primer specificity was checked using NCBI-BLAST, and by PCR using the DNA extracted from *S. elongatus (PCC 7942)*. Selective amplification of Actinobacteria was carried out using previously described specific Actinobacteria 16S rRNA primers: SC-Act-235aS20 S: 5′-CGC GGC CTA TCA GCT TGT TG-3′; SC-Act-878aA19: 5′-CCG TAC TCC CCA GGC GGG G-3′^36^.

For quantification, expression of the target gene was normalized to the expressed bacterial 16S rRNA, that was amplified using the following primers: 16S rRNA-Forward, AGAGTTTGATCCTGGCTCAG, and 16S rRNA-Reverse^36^. Colonic content wet-dry weight ratios were obtained for further normalization^37^.

### Identification of intestinal microbiota capable of generating BH4

Fecal microbiota species expressing PTPS-2 were identified by utilizing two defined microbial ecosystems. These defined ecosystems, MET-1 and MET-2, were each simplified consortia of approximately 30 microbial strains each, isolated from healthy human stool samples (1 ecosystem per individual), and were cultured in single-stage chemostats set to mimic the conditions of the distal colon^38^; briefly, a temperature of 37 °C, pH 7, and retention time of 24 hrs, using a growth medium rich in insoluble fibre and mucin^39^. The bacterial species that comprise MET-1 are described by Petrof *et al*.^15^, and those that comprise MET-2 are described by Yen *et al*.^14^. The presence of PTPS-2 in each community was determined, and those ecosystems positive for PTPS-2 expression were further examined by qPCR of their individual components to identify the specific bacterial strains.

For the determination of biopterin generation by specific Actinobacteria spp. strains, each strain was separately grown on FAA with 5% (v/v) defibrinated sheep’s blood as above for 3 days to obtain biomass to inoculate broth cultures for expansion of biomass for experiments. *M. schleiferi* 22-5-I 1_FAA_NB_aer was grown in trypticase soy broth (EMD Biosciences, Etobicoke, ON) supplemented with hemin and menadione, whereas *A. equolifaciens* 22-5-I 11_TSAB was grown in Wilkins-Chalgren broth supplemented with 0.3 g/L Cysteine HCl. Both strains were cultured for 3 days at 37 °C under anaerobic conditions (N_2_:CO_2_:H_2_, 90:5:5) in a Concept 400 anaerobe chamber (Ruskinn, UK), and the resulting culture was briefly centrifuged to pellet the bacterial cells. The bacterial pellets were separately resuspended in RNA*later*^®^ Stabilization Solution (ThermoFisher Scientific, Burlington, ON, Canada) and stored at −80 °C until further processing.

Prepared cell pellets were separately resuspended in 100 μL of 10 mM Tris-HCl, pH 8.0, 1 mM EDTA. After sonication (1 minute), each homogenate was separately mixed with an equal volume of acidic iodine solution (2% KI, 1% I_2_ (w/v) in 1 N HCl). After vortexing, the mixture was left in the dark for 1 hour at room temperature and then centrifuged at 20,000 g for 15 minutes at 4 °C. The supernatants were mixed with one-tenth volume of 5% (w/v) ascorbic acid to reduce excess iodine, and assayed by HPLC using an Ultimate 3000 Dionex system (ThermoFisher Scientific, Oakville, ON, Canada) equipped with a Kinetex™ 5 μm C18 100 Å, LC Column 150 × 4.6 mm (Phenomenex) and a fluorescence detector (Dionex RF 200 Fluorescence Detector). Biopterin was eluted with 10 mM potassium phosphate buffer (pH 6.0) at a flow rate of 0.7 mL/minute and monitored at excitation and emission wavelengths of 350 and 450 nm, respectively utilizing a commercially available standard (L-biopterin; Cedarlane, Burlington, ON, Canada). The HPLC data was acquired and processed using Chromeleon version 6.80 software (ThermoFisher Scientific, Burlington, ON, Canada).

### Statistical methods

Data were first evaluated to determine Gaussian distribution by Skewness, Kurtosis and Omnibus testing to confirm normal distribution. Data were analyzed by unpaired Student’s t-test. Statistical significance was determined at P < 0.05. All statistical analyses were performed with the Number Cruncher Statistical System software (NCSS, Kaysville, Utah, USA). Data are presented as means ± SEM.

## Additional Information

**How to cite this article**: Belik, J. *et al*. Intestinal microbiota as a tetrahydrobiopterin exogenous source in *hph-1* mice. *Sci. Rep.* **7**, 39854; doi: 10.1038/srep39854 (2017).

**Publisher's note:** Springer Nature remains neutral with regard to jurisdictional claims in published maps and institutional affiliations.

## Figures and Tables

**Table 1:** Results of screening of axenically cultured Actinobacteria spp. derived from human feces for the presence of PTPS-2.

Genus	Species	Strain	PTPS-2 expression (relative to 16 S RNA expression)*
*Actinomyces*	*odontolyticus*	NEC1 1 FAA aer	0.0
*Adlercreutzia*	*equolifaciens*	AP38TSA	4.8 ± 1.3
***Adlercreutzia***	***equolifaciens***	**22-5-I 11_TSAB**	**4.3 ± 0.1**
*Asaccharobacter*	*celatus*	UC1_BHI_P	0.0
*Asaccharobacter*	*celatus*	OB21_GAM_11_AN	0.0
*Atopobium*	*minutum*	32-6-I 11 MRS	0.0
*Atopobium*	*parvulum*	WAL 16972	0.0
*Atopobium*	*vaginae*	CC14Z	0.0
*Bifidobacterium*	*adolescentis*	AB8 #8	0.0
*Bifidobacterium*	*angulatum*	F16 #5	0.0
*Bifidobacterium*	*animalis*	AC2_8_11 AN MRS 1	0.0
*Bifidobacterium*	*bifidum*	2NTP 18OX	0.0
*Bifidobacterium*	*breve*	AP100FAA	5.6 ± 2.0
*Bifidobacterium*	*catenulatum*	NI48 #18	0.0
*Bifidobacterium*	*catenulatum*	AB8 #4	0.0
*Bifidobacterium*	*faecale*	16-6-I 20 MRS	0.0
*Bifidobacterium*	*infantis*	WAL 14599	0.0
*Bifidobacterium*	*infantis*	WAL 14760	31.9 ± 2.9
*Bifidobacterium*	*longum*	12_1_47BFAA	18.1 ± 1.4
*Bifidobacterium*	*pseudocatenulatum*	FE19 #3	1.2 ± 0.1
*Bifidobacterium*	*pseudolongum*	Wt_Minn_NB_3_AN	0.0
*Brevibacterium*	*frigoritolerans*	22-5-S 1 D5 FAA aer	0.9 ± 0.2
*Collinsella*	*aerofaciens*	4_8_47FAA	0.0
*Collinsella*	*aerofaciens*	OB21_FAA_2	0.0
*Corynebacterium*	*aurimucosum*	32-6-I 1 BHI aer	0.0
*Corynebacterium*	*singulare*	WAL 16968	0.0
*Corynebacterium*	*tuberculostearicum*	BM12Ct	0.0
*Eggerthella*	*hongkongensis*	RC2-4 B	0.0
*Eggerthella*	*hongkongensis*	CC2/3 TSA6	0.0
*Eggerthella*	*lenta*	1_3_56FAA	4.6 ± 1.1
*Eggerthella*	*lenta*	W1 BHI 6 AN	0.0
*Gordonibacter*	*urolithinfaciens*	3ASN-2LG	26.2 ± 20.1
*Gordonibacter*	*urolithinfaciens*	2NTN 15LG	0.0
***Microbacterium***	***schleiferi***	**22-5-I 1_FAA_NB_aer**	**1.9 ± 0.3**
*Micrococcus*	*luteus*	NEC1 5 TSA aer	0.0
*Mobiluncus*	*curtisii*	OB21_D5_31_AN	1.1 ± 0.1
*Olsenella*	*profusa*	OB21_FMU_23_AN	0.0
*Olsenella*	*profusa*	RC1-3 A	0.0
*Olsenella*	*uli*	AB12 #23	0.0
*Olsenella*	*uli*	A16 #8	0.0
*Synechococcus*	*elongatus*	PCC 7942	6.2 ± 0.4

^*^Mean ± SE. Strains designated in bold are isolates from the defined microbial ecosystems assessed both in chemostat culture and individually. *S. elongatus* is not a member of the Actinobacteria phylum but is included here as a reference, since it was used as a control for our assays.

## References

[b1] Crabtree M. J. & Channon K. M. Synthesis and recycling of tetrahydrobiopterin in endothelial function and vascular disease. Nitric. Oxide. 25, 81–88, doi: S1089-8603(11)00392-2 (2011). 21550412 10.1016/j.niox.2011.04.004 PMC5357050

[b2] Werner E. R., Blau N. & Thony B. Tetrahydrobiopterin: biochemistry and pathophysiology. Biochem. J. 438, 397–414, doi: BJ20110293 (2011). 21867484 10.1042/BJ20110293

[b3] Blau N., Hennermann J. B., Langenbeck U. & Lichter-Konecki U. Diagnosis, classification, and genetics of phenylketonuria and tetrahydrobiopterin (BH4) deficiencies. Molecular genetics and metabolism 104 Suppl, S2–9, doi: 10.1016/j.ymgme.2011.08.017 (2011). 21937252

[b4] Bode V. C., McDonald J. D., Guenet J. L. & Simon D. hph-1: a mouse mutant with hereditary hyperphenylalaninemia induced by ethylnitrosourea mutagenesis. Genetics 118, 299–305 (1988). 3360305 10.1093/genetics/118.2.299 PMC1203282

[b5] Belik J. . Pulmonary hypertension in the newborn GTP cyclohydrolase I-deficient mouse. Free radical biology & medicine 51, 2227–2233, doi: 10.1016/j.freeradbiomed.2011.09.012 (2011). 21982896 PMC5050525

[b6] Gutlich M. . Molecular characterization of HPH-1: a mouse mutant deficient in GTP cyclohydrolase I activity. Biochem Biophys Res Commun 203, 1675–1681, doi: 10.1006/bbrc.1994.2379 (1994). 7524491

[b7] Maeda T. . Studies on the genotype-phenotype relation in the hph-1 mouse mutant deficient in guanosine triphosphate (GTP) cyclohydrolase I activity. Brain & development 22 Suppl 1, S50–53 (2000). 10984661 10.1016/s0387-7604(00)00133-9

[b8] Welsh C., Shifrin Y., Pan J. & Belik J. Infantile hypertrophic pyloric stenosis (IHPS): A study of its pathophysiology utilizing the newborn hph-1 mouse model of the disease. American journal of physiology. Gastrointestinal and liver physiology 307, G1198–1206, doi: 10.1152/ajpgi.00221.2014 (2014). 25359537

[b9] Panteli C. New insights into the pathogenesis of infantile pyloric stenosis. Pediatr. Surg. Int. 25, 1043–1052 (2009). 19760199 10.1007/s00383-009-2484-x

[b10] Collins J., Borojevic R., Verdu E. F., Huizinga J. D. & Ratcliffe E. M. Intestinal microbiota influence the early postnatal development of the enteric nervous system. Neurogastroenterology and motility: the official journal of the European Gastrointestinal Motility Society 26, 98–107, doi: 10.1111/nmo.12236 (2014). 24329946

[b11] Chan Y. K., Estaki M. & Gibson D. L. Clinical consequences of diet-induced dysbiosis. Annals of nutrition & metabolism 63 Suppl 2, 28–40, doi: 10.1159/000354902 (2013). 24217034

[b12] Anitha M., Vijay-Kumar M., Sitaraman S. V., Gewirtz A. T. & Srinivasan S. Gut microbial products regulate murine gastrointestinal motility via Toll-like receptor 4 signaling. Gastroenterology 143, 1006–1016 e1004, doi: 10.1053/j.gastro.2012.06.034 (2012). 22732731 PMC3458182

[b13] Kong J. S. . 6-Pyruvoyltetrahydropterin synthase orthologs of either a single or dual domain structure are responsible for tetrahydrobiopterin synthesis in bacteria. FEBS letters 580, 4900–4904, doi: 10.1016/j.febslet.2006.08.006 (2006). 16920111

[b14] Yen S. . Metabolomic analysis of human fecal microbiota: a comparison of feces-derived communities and defined mixed communities. J Proteome Res 14, 1472–1482, doi: 10.1021/pr5011247 (2015). 25670064

[b15] Petrof E. O. . Stool substitute transplant therapy for the eradication of Clostridium difficile infection: ‘RePOOPulating’ the gut. Microbiome 1, 3, doi: 10.1186/2049-2618-1-3 (2013). 24467987 PMC3869191

[b16] Goldsmith F., O’Sullivan A., Smilowitz J. T. & Freeman S. L. Lactation and Intestinal Microbiota: How Early Diet Shapes the Infant Gut. J Mammary Gland Biol Neoplasia 20, 149–158, doi: 10.1007/s10911-015-9335-2 (2015). 26227402

[b17] Underwood M. A., German J. B., Lebrilla C. B. & Mills D. A. Bifidobacterium longum subspecies infantis: champion colonizer of the infant gut. Pediatr Res 77, 229–235, doi: 10.1038/pr.2014.156 (2015). 25303277 PMC4350908

[b18] Hyland K., Gunasekara R. S., Munk‐Martin T. L., Arnold L. A. & Engle T. The hph‐1 mouse: A model for dominantly inherited GTP‐cyclohydrolase deficiency. Annals of neurology 54, S46–S48 (2003). 12891653 10.1002/ana.10695

[b19] Hyland K., Gunasekera R. S., Engle T. & Arnold L. A. Tetrahydrobiopterin and biogenic amine metabolism in the hph-1 mouse. J. Neurochem. 67, 752–759 (1996). 8764604 10.1046/j.1471-4159.1996.67020752.x

[b20] McDonald J. & Bode V. Hyperphenylalaninemia in the hph-1 mouse mutant. Pediatr. Res. (1988). 10.1203/00006450-198801000-00014 3340448

[b21] Peeters B., Benninga M. A. & Hennekam R. C. M. Infantile hypertrophic pyloric stenosis–genetics and syndromes. Nat Rev Gastroenterol Hepatol 9, 646–660 (2012). 22777173 10.1038/nrgastro.2012.133

[b22] Crabtree M. J., Hale A. B. & Channon K. M. Dihydrofolate reductase protects endothelial nitric oxide synthase from uncoupling in tetrahydrobiopterin deficiency. Free Radic. Biol. Med. 50, 1639–1646 (2011). 21402147 10.1016/j.freeradbiomed.2011.03.010 PMC3121954

[b23] Rolfe V. E., Brand M. P., Heales S. J., Lindley K. J. & Milla P. J. Tetrahydrobiopterin regulates cyclic GMP-dependent electrogenic Cl- secretion in mouse ileum *in vitro*. J. Physiol. (Lond.) 503 (Pt 2), 347–352 (1997). 9306277 10.1111/j.1469-7793.1997.347bh.x PMC1159867

[b24] Forrest H. S. & Van B. C. Microbiology of unconjugated pteridines. Annu. Rev. Microbiol. 24, 91–108 (1970). 4927141 10.1146/annurev.mi.24.100170.000515

[b25] Urushibara T., Forrest H. S., Hoare D. S. & Patel R. N. Pteridine content of some methane- and methanol-oxidizing bacteria. Experientia 28, 392–393 (1972). 4556500 10.1007/BF02008294

[b26] Tiso M. & Schechter A. N. Nitrate Reduction to Nitrite, Nitric Oxide and Ammonia by Gut Bacteria under Physiological Conditions. (2015). 10.1371/journal.pone.0119712 PMC4372352 25803049

[b27] Hezel M. P. & Weitzberg E. The oral microbiome and nitric oxide homoeostasis. Oral Dis 21, 7–16, doi: 10.1111/odi.12157 (2015). 23837897

[b28] Strozzi G. P. & Mogna L. Quantification of folic acid in human feces after administration of Bifidobacterium probiotic strains. J. Clin. Gastroenterol. 42 Suppl 3 Pt 2, S179–S184 (2008). 18685499 10.1097/MCG.0b013e31818087d8

[b29] Aufreiter S., Kim J. H. & O’Connor D. L. Dietary oligosaccharides increase colonic weight and the amount but not concentration of bacterially synthesized folate in the colon of piglets. J. Nutr. 141, 366–372 (2011). 21270368 10.3945/jn.110.135343

[b30] Rossi M., Amaretti A. & Raimondi S. Folate production by probiotic bacteria. Nutrients. 3, 118–134 (2011). 22254078 10.3390/nu3010118 PMC3257725

[b31] Burnett J. R. Sapropterin dihydrochloride (Kuvan/phenoptin), an orally active synthetic form of BH4 for the treatment of phenylketonuria. IDrugs: the investigational drugs journal 10, 805–813 (2007). 17968763

[b32] Turroni F. . Diversity of bifidobacteria within the infant gut microbiota. PLoS. One. 7, e36957 (2012). 22606315 10.1371/journal.pone.0036957 PMC3350489

[b33] Weinmann A. . Tetrahydrobiopterin is present in high quantity in human milk and has a vasorelaxing effect on newborn rat mesenteric arteries. Pediatr Res 69, 325–329, doi: 10.1203/PDR.0b013e31820bc13a (2011). 21178821

[b34] Krogh C. . Bottle-feeding and the Risk of Pyloric Stenosis. Pediatrics 130, e943–949 (2012). 22945411 10.1542/peds.2011-2785 PMC3457615

[b35] Arning E. & Bottiglieri T. LC-MS/MS Analysis of Cerebrospinal Fluid Metabolites in the Pterin Biosynthetic Pathway. JIMD reports, doi: 10.1007/8904_2014_336 (2014). PMC5059177 25213568

[b36] Stach J. E., Maldonado L. A., Ward A. C., Goodfellow M. & Bull A. T. New primers for the class Actinobacteria: application to marine andĲÜ terrestrial environments. Environmental microbiology 5, 828–841 (2003). 14510836 10.1046/j.1462-2920.2003.00483.x

[b37] Krause L. J., Forsberg C. W. & O’Connor D. L. Feeding human milk to rats increases Bifidobacterium in the cecum and colon which correlates with enhanced folate status. J. Nutr. 126, 1505–1511 (1996). 8618150 10.1093/jn/126.5.1505

[b38] McDonald J. A. . Simulating distal gut mucosal and luminal communities using packed-column biofilm reactors and an *in vitro* chemostat model. Journal of microbiological methods 108, 36–44, doi: 10.1016/j.mimet.2014.11.007 (2015). 25462016

[b39] McDonald J. A. . Evaluation of microbial community reproducibility, stability and composition in a human distal gut chemostat model. Journal of microbiological methods 95, 167–174, doi: 10.1016/j.mimet.2013.08.008 (2013). 23994646

