# Corrigendum: Intestinal microbiota as a tetrahydrobiopterin exogenous source in *hph*-*1* mice

**DOI:** 10.1038/srep44161

**Published:** 2017-04-11

**Authors:** Jaques Belik, Yulia Shifrin, Erland Arning, Teodoro Bottiglieri, Jingyi Pan, Michelle C. Daigneault, Emma Allen-Vercoe

Scientific Reports 7: Article number: 3985410.1038/srep39854; published online: 01 12 2017; updated: 04 11 2017

In the original version of this Article, all instances of “*Adlercreutzia equolifaciens*” were incorrectly given as “*Aldercreutzia equolifaciens*”. This error has now been corrected in the PDF and HTML versions of the Article.

